# Human Intestinal Enteroids Model MHC-II in the Gut Epithelium

**DOI:** 10.3389/fimmu.2019.01970

**Published:** 2019-08-20

**Authors:** Jonathan E. Wosen, Alexandra Ilstad-Minnihan, Julia Y. Co, Wei Jiang, Dhriti Mukhopadhyay, Nielsen Q. Fernandez-Becker, Calvin J. Kuo, Manuel R. Amieva, Elizabeth D. Mellins

**Affiliations:** ^1^Program in Immunology, Department of Pediatrics, Stanford University, Stanford, CA, United States; ^2^Division of Infectious Diseases, Department of Pediatrics, Stanford University, Stanford, CA, United States; ^3^Division of Gastroenterology and Hepatology, Department of Medicine, Stanford University, Stanford, CA, United States; ^4^Division of Hematology, Department of Medicine, Stanford University, Stanford, CA, United States; ^5^Department of Microbiology and Immunology, Stanford University, Stanford, CA, United States

**Keywords:** MHC-II, epithelial cells, enteroid culture, antigen presentation, mucosal immunity

## Abstract

The role of intestinal epithelial cells (IECs) in mucosal tolerance and immunity remains poorly understood. We present a method for inducing MHC class II (MHC-II) in human enteroids, “mini-guts” derived from small intestinal crypt stem cells, and show that the intracellular MHC-II peptide-pathway is intact and functional in IECs. Our approach enables human enteroids to be used for novel *in vitro* studies into IEC MHC-II regulation and function during health and disease.

## Introduction

Intestinal epithelial cells (IECs) are well-positioned to interact with both the gut microbial and dietary milieu as well as the underlying mucosal immune system. Numerous prior reports show that IECs constitutively express Major Histocompatibility Complex II (MHC-II), a key molecule in the stimulation of CD4+ T cell responses (1–3). This raises the possibilities that IECs contribute to tolerance and immunity through MHC-II-mediated antigen presentation and that IEC MHC-II is involved in intestinal disorders, such as inflammatory bowel and celiac disease. A recent study demonstrates that, in mice, MHC-II expressed by intestinal stem cells allows for immune-epithelial cross-talk that modulates epithelial renewal and differentiation (4). The role of IEC MHC-II in humans, however, remains poorly understood due to limited IEC model systems. Standard colonic cell lines—including T84, Caco-2, and HT29—are problematic because MHC-II is expressed in the small intestine, but not the colonic epithelium under non-inflammatory conditions (5). Additionally, these adenocarcinoma-derived cell lines possess epigenetic modifications known to alter MHC-II expression (6).

In recent years, long-lived primary cell-derived cultures have emerged as an attractive model system for the gut epithelium. These cultures, known as organoids when derived from pluripotent stem cells or enteroids when grown from adult stem cells, recapitulate critical gut epithelial structures and functions, including polarization and brush border formation, intestinal stem cell renewal, and differentiation into specialized epithelial cell types (7, 8). Previous studies demonstrate MHC-II can be induced in mouse enteroids, but human enteroids have not yet been tested (9). We report the first protocol for the induction of MHC-II in human intestinal enteroids. We based our approach on the previously demonstrated finding that MHC-II is transcriptionally regulated by the master regulator Class II Transactivator (CIITA), and its pIV isoform controls constitutive MHC-II expression in thymic epithelium and interferon gamma-inducible expression in other non-hematopoietic cells (10, 11). IEC MHC-II is likely maintained by interferon gamma (IFNg) produced by lamina propria immune cells and intraepithelial lymphocytes in response to both microbial and systemic signals (12). Because *in vivo* MHC-II expression is restricted to the small intestine epithelium at baseline, we grew small intestinal enteroids and generated cultures of pure epithelium, as determined by flow cytometry staining for the epithelial marker EPCAM and the absence of pan-immune marker CD45 (Supplementary Figure 1). We find that the intracellular MHC-II peptide-pathway is intact and functional in IECs, providing a system to test, for example, their potential role in antigen presentation.

## Materials and Methods

### Enteroid Culture

Biopsy-derived human duodenal and ileal enteroids were established using protocols described elsewhere (7, 13). De-identified tissue was obtained with patient consent and all experiments were approved by the Stanford University Medical Center Institutional Review Board and performed under protocol #28908. Briefly, biopsy tissue from healthy adults with no defined intestinal pathology (IRB-28908) was digested in 2 mg/mL of type 1 collagenase (Fisher Scientific) at 37°C, with vigorous pipetting to dislodge crypts. Crypts were centrifuged and resuspended in Basement Membrane Extract Type 2 (Trevigen) and seeded onto a 24-well plate. Enteroids were grown in conditioned media produced by the supportive cell line L-WRN to supply Wnt, R-spondin and noggin (14). Conditioned media was supplemented with 1M HEPES (Thermo Fisher), 1× Glutamax (Thermo Fisher), 10 μM Y-27632 (Sigma-Aldrich), 50 ng/ml EGF (Thermo Fisher), 1× B27(Thermo Fisher), 10 μM SB202190 (Sigma-Aldrich), 2.5 μM CHIR99021 (Stem Cell Technologies), 500 nM A-83-01(Tocris), and 1× Normocin (InvivoGen), in accordance with established protocols (15, 16). To induce differentiation, enteroids were grown for 2 days without Wnt or R-spondin and 5 μM of gamma-secretase inhibitor DAPT was added (Sigma-Aldrich). Enteroid media was refreshed every 2 days and enteroids were passaged weekly. During passaging, TrypLE Express (Thermo Fisher) was added to enteroids and incubated at 37°C for 5 min to generate multicellular fragments for expansion. Enteroids were vigorously pipetted and enteroid fragments resuspended in fresh Basement Membrane Extract.

### Interferon Gamma Treatment

To induce MHC-II, recombinant human interferon gamma (PeproTech) was added to enteroid media at the indicated dose and length of time. For IFNg blockade, the indicated dose of anti-human IFNg antibody (clone B27, Biolegend) was added at the same time as IFNg.

### Image Analysis

Enteroids were fixed in 2% paraformaldehyde for 1 h at room temperature. Samples were then washed twice with Phosphate Buffed Saline (PBS) and stained whole-mount overnight at room temperature in a PBS solution containing 3% Bovine Serum Albumin and 1% Saponin. To detect HLA-DR, enteroids were stained with 10 μg/ml of the pan-DR monoclonal antibody, L243, conjugated to Fluorescein Isothiocyanate (FITC) (BioLegend). LAMP1 was detected with 10 μg/ml of monoclonal antibody H4A3 conjugated to Alexa Fluor 488 (BioLegend) and EEA1 was detected with 10 μg/ml of monoclonal antibody clone 14 conjugated to FITC (BD Biosciences). Goblet cells were detected with 2 μg/ml of polyclonal rabbit anti-MUC2 (Santa Cruz) in a PBS solution containing 3% Bovine Serum Albumin, 1% Saponin, and 1% Triton X-100. Samples were then PBS washed and stained with a 1:400 dilution of DAPI (Thermo Fisher) and a 1:40 dilution of Alexa Fluor 660 Phalloidin (Thermo Fisher) for 2–3 h at room temperature. For goblet cell staining, 5 μg/ml of goat anti-rabbit Alexa Fluor 488 secondary (Thermo Fisher) was added at the same time as DAPI and Phalloidin. Samples were then mounted in VectaShield Antifade Mounting Medium (Vector Labs) and imaged on a Zeiss LSM 700 confocal microscope. Image analysis was conducted with Volocity software (Improvision, Santa Clara, CA).

For tissue staining, tissue embedded in Optimal Cutting Temperature (OCT) was frozen at −80°C and 10 micron sections generated with a Leica CM1950 cryostat (Leica Biosystems). For MHC-II staining, tissue sections were washed in PBS and blocked in a PBS solution containing 3% Bovine Serum Albumin and 1% Saponin for 1 h at room temperature. Samples were stained with 10 μg/ml L243-FITC overnight at 4°C, washed with PBS, and then stained with 1:400 DAPI and 1:40 Alexa Fluor 660 Phalloidin for 1 h at room temperature before confocal imaging. For IFNGR1 staining, tissue sections were blocked in a PBS solution containing 1% Bovine Serum Albumin and stained overnight in 0.1% Bovine Serum Albumin containing 16.8 μg/ml polyclonal rabbit anti-IFNGR1 (Thermo Fisher) followed by 5 μg/ml of goat anti-rabbit Alexa Fluor 488 secondary (Thermo Fisher).

### Flow Cytometry

Enteroids were digested in TrypLE Express for 15 min in a 37°C water bath to generate a single cell suspension. TrypLE Express was used as opposed to trypsin in order to preserve surface epitopes and in accordance with previous reports (15). Samples were then resuspended in a minimum 200 μl of blocking buffer of PBS plus 5% Heat Inactivated Human AB Serum (Mediatech), 5% Normal Goat Serum (Thermo Fisher), and 10 μM Y-27632. Cells were live-dead stained for 15 min on ice using 1 μl of LIVE/DEAD Fixable Aqua Dead Cell Stain (Thermo Fisher) per 50 μl of blocking buffer. For surface HLA-DR staining, samples were stained with 5 μg/ml of APC/Cy7 HLA-DR antibody (clone L243, Biolegend) or isotype control (clone MOPC-173, BioLegend). Surface HLA-DQ staining was performed with 2.5 μg/ml of PE-conjugated pan-DQ antibody (clone 1a3, Leinco Technologies) or IgG2a kappa isotype control (eBioscience). HLA-DP was stained indirectly with 10 μg/ml of B7/21 antibody (17) produced in ascites fluid (also commercially available from Abcam and Leinco Technologies), followed by 2 μg/ml of Brilliant Violet 605 Goat anti-Mouse secondary antibody (BioLegend).

For surface CLIP staining, cells were stained with a 1:5 dilution of FITC conjugated CerCLIP.1 (BD Biosciences) (18). SPVL3, a mouse antibody that recognizes HLA-DQ in a DM-dependent manner, was also used (1:50 dilution) followed by 2 μg/ml of APC Goat anti-Mouse secondary (BD Pharmingen). To confirm purity of intestinal epithelial cultures, samples were stained with 5 μg/ml FITC CD45 (clone H130, BioLegend) and a 1:200 dilution of APC EPCAM (clone 9c4, BioLegend). IFNGR1 was measured with a 1:20 dilution of mouse anti-IFNGR1 (Caltag) followed by staining with a 1:100 dilution of goat anti-mouse Brilliant Violet 605 secondary antibody (BioLegend). All surface staining was performed in blocking buffer for 30 min on ice prior to fixation with BD Cytofix (BD Biosciences) for 15 min on ice. For intracellular staining, samples were treated with BD Cytofix immediately after live-dead staining and permeabilized in BD Perm/Wash (BD Biosciences) for 15 min at room temperature. Samples were then stained in Perm/Wash for 30 min on ice with 7.5 μg/ml of Alexa Fluor 647 Map.DM1 (19) for HLA-DM detection, or a 1:250 dilution of FITC Mags.DO5 (20) for HLA-DO detection. Following staining and fixation, samples were resuspended in PBS with 0.5% Bovine Serum Albumin and run on a Cytek DPX10 FACS machine (Cytek Biosciences). Isotype controls, fluorescence minus one (FMO), secondary only, and biological positive and negative controls were used where appropriate. Data analysis was performed using FlowJo software (Tree Star, Ashland, OR).

### Cell Viability Assay

To assess enteroid viability, 50 μl alamarBlue (Thermo Fisher) was added to 500 μl of media per well of actively growing enteroids and incubated overnight at 37°C. Fluorescence was then measured on a Tecan microplate reader (Tecan Life Sciences), with an excitation wavelength of 570 nm and emission detected at 585 nm.

### Enteroid Polarity Reversal

A detailed protocol for enteroid polarity reversal is described elsewhere (21). Briefly, enteroids were resuspended in Cell Recovery Solution (Fisher Scientific) and placed on a rocker at 4°C for 1.5 h to remove Basement Membrane Extract (BME). Samples were washed with PBS and resuspended in cold enteroid media or media supplemented with 7.5% soluble BME to generate apical-out and basolateral-out enteroids, respectively. Enteroids were then plated in a Corning® Costar® Ultra-Low Attachment 24-well plate (Sigma Aldrich).

### Barrier Function Assay

Basolateral-out enteroids grown in suspension (7.5% BME) were treated with 100 U/ml of IFNg for 72 h or left untreated. Enteroids were then incubated in 2 mg/ml of 4 kDa FITC-Dextran and live imaged with differential interference contrast and fluorescence microscopy. Enteroids resuspended in 2 mM EDTA in magnesium- and calcium-free PBS were used as a positive control.

### Monolayer Formation

Seventy-five microliter of BME diluted 1:40 in ice-cold PBS were added to the top membrane of a 6.5 mm 24-well plate transwell with a 0.4 μm pore insert (Corning), transferred to a 37°C incubator for 1 h, and then washed three times in PBS. Enteroids were then digested to single cells with TrypLE Express and added to the upper chamber of the transwell membrane. Six hundred microliter of enteroid culture media was added to the lower chamber. For MHC-II induction, 100 U/ml of IFNg was added to the lower chamber (basolateral induction) and monolayers were cultured for an additional 72 h.

### Western Blot

Enteroids were resuspended in Cell Recovery Solution (Fisher Scientific) and placed on a rocker at 4°C for 1.5 h to remove Basement Membrane Extract (BME). Samples were then washed in PBS and lysed with ice-cold RIPA buffer, mixed with reducing SDS sample buffer, and boiled. SDS-PAGE was performed with a 4–12% Criterion Bis-Tris Protein Gel (BioRad) at 145 V for 55 min and protein transferred to a nitrocellulose membrane. Samples were blocked with SuperBlock T20 Blocking Buffer (Thermo Fisher) and probed with DA6.147, a mouse monoclonal antibody that recognizes HLA-DR alpha (22), diluted 1:1000 from ascites, followed by a 1:5000 dilution of HRP-conjugated horse anti-mouse secondary (Cell Signaling Technology). Super Signal West Femto Maximum Sensitivity Substrate (Thermo Fisher) was applied and the blot was imaged on a chemiluminescent imaging system (Azure Biosystems). The blot was then stripped and probed with HRP-conjugated mouse anti-beta actin diluted 1:5000 (Cell Signaling Technology) before substrate addition and imaging.

### qRT-PCR

RNA was extracted from enteroids via a TRIzol/RNeasy hybrid extraction protocol. Briefly, enteroids were initially digested in TRIzol (Thermo Fisher) and processed through a standard phenol-chloroform extraction. After aqueous phase removal, samples were eluted in ethanol, transferred to an RNeasy column (Qiagen), and processed with a RNeasy Mini Kit (Qiagen) to obtain RNA. RNA was reverse transcribed into cDNA using iScript™ Reverse Transcription Supermix (BioRad) and PCR performed with SsoAdvanced™ Universal SYBR® Green Supermix (BioRad) on a C1000 Thermal Cycler (BioRad). GAPDH was used as a housekeeping gene. For HLA-DQA, commercially available primers were used (PrimePCR Assay HLA-DQA1 primers, BioRad). See Table 1 for all other primer sequences. Primers were used at 10 μM, and fold changes were calculated with the delta-delta Ct method, based on the equation below.

$$\begin{array}{l} Fold\;Change\\ \;=\;2^{\left(target\;gene\;control-target\;gene\;treated\right)-\left(GAPDH\;control-GAPDH\;treated\right)} \end{array}$$

**Table 1:** Primer sequences.

GAPDH-F	5^′^- GACCTGCCGTCTAGAAAAACC
GAPDH-R	5^′^- GCTGTAGCCAAATTCGTTGTC
HLA-DRA-F	5^′^-TGGAGTCCCTGTGCTAGGAT
HLA-DRA-R	5^′^-ATAGAACTCGGCCTGGATGA
HLA-DRB-F	5^′^-AGTGACACTGATGGTGCTGAG
HLA-DRB-R	5^′^-TCCGTCCCATTGAAGAAATG
HLA-DPA-F	5^′^-CTTGGCTTTCCTGCTGAGTC
HLA-DPA-R	5^′^-CCCTGTTGGTCTATGCGTCT
Invariant Chain-F	5^′^-CGCGACCTTATCTCCAACA
Invariant Chain-R	5^′^-CAGGATGGAAAAGCCTGTGT
TLR4-F	5-GGACTCTGATCCCAGCCAT
TLR4-R	5^′^-TGCCCCATCTTCAATTGTCT
CXCL9-F	5^′^-CCTTAAACAATTTGCCCCAA
CXCL9-R	5^′^-TCACATCTGCTGAATCTGGG
CXCL10-F	5^′^-CACCATGAATCAAACTGCGA
CXCL10-R	5^′^-GCTGATGCAGGTACAGCGT
ALPI-F	5^′^-TACACGTCCATCCTGTACGG
ALPI-R	5^′^-CTCGCTCTCATTCACGTCTG
LGR5-F	5^′^-CCTTCATAAGAAAGATGCTGGAAT
LGR5-R	5^′^-GTTTAATGGGGGAAATGTACAGAG
MUC2-F	5^′^-AGGATCTGAAGAAGTGTGTCACTG
MUC2-R	5^′^-TAATGGAACAGATGTTGAAGTGCT
LYZ-F	5^′^-GGTTACAACACACGAGCTACAAAC
LYZ-R	5^′^-AGTTACACTCCACAACCTTGAACA
CHGA-F	5^′^-AGAATTTACTGAAGGAGCTCCAAG
CHGA-R	5^′^-TCCTCTCTTTTCTCCATAACATCC

## Results

### IFNg Induces all Three MHC-II Proteins in Enteroids

**Figure 1:**
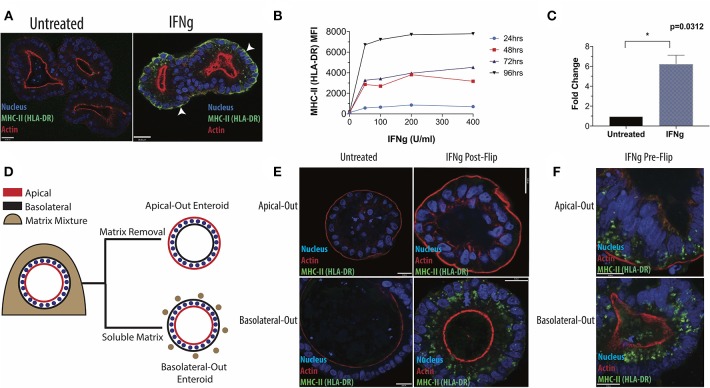
Induction of HLA-DR in human small intestinal enteroids. **(A)** Enteroids were stained with antibody to the MHC-II protein, HLA-DR, under standard culture conditions or following IFNg treatment (100 U/ml, 72 h) and imaged via fluorescence microscopy. White arrowheads indicate basolateral HLA-DR. **(B)** Median fluorescence intensity (MFI) of surface HLA-DR levels on enteroids treated for 24, 48, 72, or 96 h with 0–400 U/ml IFNg and analyzed by flow cytometry. Data are representative of two experiments. **(C)** RT-qPCR quantification of HLA-DR beta transcript in six enteroid lines, with GAPDH as a housekeeping gene. Error bars correspond to standard error of the mean. Statistical comparison of IFNg-treated and untreated enteroids performed by Wilcoxon Matched Pairs Signs Rank test. **(D)** Diagram of enteroid polarity reversal, whereby removal of extracellular matrix scaffold induces enteroids to adopt an apical-out morphology. **(E)** Apical-out and basolateral-out enteroids were treated with IFNg and imaged for HLA-DR or were pre-treated with IFNg prior to polarity reversal **(F)**. Images in **(E,F)** are representative of three experiments. Scale bar = 20 μm. ^*^p < 0.05.

To evaluate whether human small intestinal enteroids had the machinery necessary for response to IFNg, we determined by flow cytometry that the ligand-binding subunit of IFNg receptor, IFNGR1, was present (Supplementary Figure 1f). We then showed that treatment with IFNg induced MHC-II protein HLA-DR both intracellularly and along the cell membrane, as visualized by immunofluorescence microscopy (Figure 1A). IFNg also induced the MHC-II proteins HLA-DP and HLA-DQ at the transcript and protein level, as detected by RT-qPCR and flow cytometry, respectively (Supplementary Figure 2). We noted donor-to-donor variability MHC-II protein levels and tested the possibility that variation in IFNGR1 levels was responsible. Relative levels of MHC-II in our panel of patient-derived enteroids remain consistent across induction experiments. Although all lines express surface IFNGR1, IFNGR1 levels do not correlate with induced MHC-II levels, suggesting that variation in IFNGR1 expression alone does not drive variation in enteroid MHC-II expression (Supplementary Figure 2e).

In subsequent experiments, we focused on HLA-DR, which was readily detectible by microscopy. IFNg treatment induced HLA-DR in both a dose- and time-dependent manner, detected by flow cytometry (Figure 1B), and up-regulated DRβ transcript, as measured by qPCR (Figure 1C). In the native intestine, IECs stain strongly for HLA-DR along their lateral and basolateral surfaces (Supplementary Figure 3). Therefore, we varied the conditions of IFNg treatment to more closely recapitulate the *in vivo* distribution of HLA-DR. We find that prolonged IFNg exposure significantly increases the relative proportion of surface HLA-DR, as shown by flow cytometry (Supplementary Figure 4a). Fluorescence microscopy shows that prolonged IFNg treatment leads to increased MHC-II along the basolateral surface, where IECs and gut immune cells interact *in vivo* (Supplementary Figure 4b). These observations suggest that the *in vivo* distribution of HLA-DR is likely the result of prolonged IEC exposure to IFNg from resident immune cells.

Researchers seeking to use our protocol to evaluate the effects of IEC MHC-II on immune cell activation and maturation will likely want to avoid confounding effects of IFNg exposure on immune cell activity. We therefore tested whether MHC-II expression could be maintained in IFNg-free media by treating enteroids with IFNg for 3 days and tracking HLA-DR levels. HLA-DR remained detectable up to 6 days after switching enteroids to IFNg-free media, with reduced levels by 4 days (Supplementary Figures 5a,b). Our findings suggest that short-term enteroid-immune cell co-cultures to evaluate the effects of IEC MHC-II are feasible. We also simultaneously treated enteroids with IFNg and an anti-IFNg blocking antibody and observed a dose-dependent reduction in MHC-II induction detected by flow cytometry (Supplementary Figure 5c). This finding further demonstrates the quantitative effects of IFNg on induction of IEC MHC-II.

### IFNg-Treated Enteroids Exhibit Reduced Stemness, but Maintain Viability and Barrier Integrity

As IFNg is known to have pleiotropic effects, we also tested IFNg-treated enteroids for non-MHC related phenotypes. We tested various doses and times of IFNg treatment and found that, under the conditions tested, IFNg treatment had no effect on cell viability or barrier function, in contrast to previous findings in mouse enteroids [Supplementary Figure 6; (23)]. Notably, IFNg treatment led to downregulation of LGR5 (*p* = 0.0417), an intestinal stem cell marker, and a modest increase in alkaline phosphatase, a marker of mature enterocytes, that was not significant (*p* = 0.1562) (Supplementary Figure 7a). Markers for goblet and enteroendocrine cells were not significantly affected by IFNg treatment, though we observed a reduction in lysozyme, a Paneth cell marker, at near statistical significance (*p* = 0.0781). IFNg-induced downregulation of intestinal stem cell and Paneth cell markers, which has been reported previously, suggests a link between MHC-II expression and the transition of IECs from an undifferentiated, stem-cell state to a more mature state (23). We therefore tested whether differentiation was sufficient to induce MHC-II. Standard enteroid culture media contains high levels of Wnt and R-spondin, which promote intestinal stem cell maintenance, but withdrawal of these factors induces epithelial differentiation. Differentiated enteroids did not express HLA-DR protein, as detected by western blot (Supplementary Figure 8a), but differentiation elevated HLA-DR alpha and beta subunit transcripts (Supplementary Figure 8b). The absence of HLA-DR protein in differentiated enteroids was further confirmed by flow cytometry staining in comparison to T2, an MHC-II negative T cell/B cell hybrid line (Supplementary Figure 8c), and microscopy [Supplementary Figure 8d; (24)]. This suggests that epithelial differentiation is insufficient to fully induce MHC-II, but that differentiated IECs may be better able to express MHC-II in response to IFNg. Further work is needed to explore this possibility, but these observations may help explain the *in vivo* expression of MHC-II in the mature enterocytes of the villus and its absence in the intestinal stem cells of the crypt at homeostasis (Supplementary Figure 3).

We also detected robust increases in expression of known interferon stimulated genes, such as chemokines CXCL9 and CXCL10, and a modest but significant increase in TLR4 (Supplementary Figure 7b). CXCL9 and CXCL10 are key regulators of T cell trafficking, and elevated CXCL10 promotes T cell recruitment in celiac disease (25). Previous studies have shown that IFNg treatment increases IEC responsiveness to LPS via TLR4 upregulation (26). Our findings suggest that MHC-II expressing enteroids generated through our protocol may also be used to study IEC regulation of lymphocyte recruitment and IEC responsiveness to inflammatory stimuli. More work is needed to better characterize these and other known effects of IFNg [Reviewed in (27)].

### Enteroid MHC-II Induction Requires Basolateral IFNg

Next, we adapted our protocol to facilitate studies into the role of IEC polarity on MHC-II regulation and function. Enteroids typically form three-dimensional spheroids with the apical surface facing the lumen and the basolateral surface facing outward. This makes the apical surface difficult to access without microinjection, which is time-intensive and low-throughput, or monolayer formation, which requires large cell numbers. Instead, we reversed enteroid polarity by removing the extracellular matrix scaffold in which enteroids are typically grown (21). Matrix removal causes enteroids to reverse their polarity such that their apical surface faces outwards, whereas addition of 7.5% soluble matrix components to the media maintains basolateral-out polarity (Figure 1D). We exposed IECs to IFNg from the apical or basolateral surface, using apical-out and basolateral-out enteroids, respectively. After IFNg treatment, apical-out enteroids lacked MHC-II, in contrast to clear induction in basolateral-out enteroids (Figure 1E), indicating that enteroid MHC-II expression requires IFNg from the basolateral route of exposure, as would occur *in vivo* as IECs interact with lamina propria immune cells along their basolateral surface. We also reversed the polarity of enteroids after treatment with IFNg and found that MHC-II expression and localization were maintained (Figure 1F). For comparison, we treated enteroid monolayers with IFNg from the basolateral surface and also successfully induced MHC-II (Supplementary Figure 9). The dependence of enteroids on basolateral IFNg for MHC-II induction is consistent with the localization of IFNGR1 to the basal pole of IECs in villus and crypt epithelium (Supplementary Figure 10). Basal IFNGR1 localization has been reported in airway epithelium, where MHC-II can also be induced by IFNg (28).

### The MHC-II Peptide Pathway Is Functional in Enteroids

**Figure 2:**
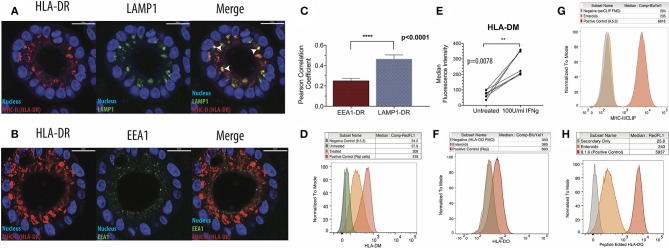
Intestinal epithelial cells possess functional MHC-II compartments. **(A)** Enteroids were stained for HLA-DR, LAMP1 (late endosome/lysosome marker), and EEA1 (early endosome marker). Representative immunofluorescence images show patterns of HLA-DR (left) and LAMP1 (middle) expression, with arrowheads indicating regions of colocalization (right; merge) and **(B)** patterns of HLA-DR (left) and EEA1 (middle) expression and merged images (right); scale bar = 20 μm. **(C)** Summary of image quantification comparing LAMP1-HLA-DR and EEA1-HLA-DR colocalization, using Volocity cellular analysis and imaging software. Eight biological replicates were measured, and data were analyzed for significance by Mann Whitney test. Error bars correspond to standard error of the mean. **(D,E)** Representative histograms **(D)** and summary of data **(E)** on expression of HLA-DM using flow cytometry. In **(E)**, data were analyzed for significance with a Wilcoxon Matched Pairs Signs Rank test. **(F)** Measurement of HLA-DO expression in IFNg-treated enteroids by flow cytometry, representative of three biological replicates. A DM+, DO+ B cell lymphoblastoid cell line (B-LCL; Raji) was used as a positive control. FMO = Fluorescence Minus One negative control. **(G)** Enteroids stained for surface CLIP with the cerCLIP.1 antibody. A DM-null B-LCL (9.5.3) was used as a positive control. **(H)** Enteroids stained with SPVL3, an antibody that recognizes an HLA-DM-dependent confirmation of HLA-DQ. A DM-competent B-LCL (8.1.6) was used as a positive control. Data in **(G,H)** are representative of three biological replicates. ^**^p < 0.01, ^****^p < 0.0001.

Our next goal was to evaluate whether enteroids were equipped to efficiently present antigen. In antigen presenting cells, newly synthesized MHC-II associates with invariant chain (Ii), a dedicated chaperone protein that directs MHC-II into late-stage endosomal compartments known as MHC-II compartments (MIICs). A region of full-length Ii occupies the peptide binding groove of MHC-II. Proteases within the MIIC cleave Ii and leave a nested set of invariant chain fragments known as class II invariant chain-associated peptides (CLIP) bound to the grooves of MHC-II molecules. CLIP is then exchanged in favor of high-affinity peptide binders in a process catalyzed by HLA-DM, a non-classical MHC protein (29). Some studies show that HLA-DM and MHC-II do not colocalize in non-professional antigen presenting cells, suggesting that these cells do not form an MIIC (30). To detect whether IECs possess an MIIC, IFNg-treated enteroids were co-stained for HLA-DR and LAMP1 or EEA1, markers of late and early endosomes, respectively (Figures 2A,B). MHC-II preferentially colocalized with LAMP1, confirmed by image quantification, demonstrating trafficking of MHC-II into late endosomes (Figure 2C).

After confirming Ii expression (Supplementary Figure 4c), we then investigated whether HLA-DM was present and active in the enteroids. HLA-DM was absent prior to treatment, but induced in response to IFNg (Figures 2D,E). Notably, HLA-DO, a negative regulator of HLA-DM detected in thymic medullary epithelial cells, was absent (Figure 2F). To test HLA-DM activity, we measured surface levels of CLIP; when HLA-DM is functional, surface CLIP levels are low. Using a CLIP-specific monoclonal antibody, cerCLIP.1, to detect CLIP bound to MHC-II, we found that CLIP was nearly absent on the cell surface (Figure 2G). In contrast, 9.5.3, an HLA-DM-negative B cell lymphoblastoid line used as a control, had markedly higher surface and intracellular CLIP levels, indicative of defective HLA-DM peptide editing. To provide further evidence for peptide editing, we stained enteroids with SPVL3, an HLA-DM-dependent antibody that recognizes HLA-DQ (31). We observed strong SPVL3 staining, which is consistent with HLA-DM-mediated removal of CLIP from HLA-DQ (Figure 2H). MHC-II molecules require peptide binding groove occupancy for stabilization; MHC-II molecules on the enteroid surface are likely loaded with intracellular and/or culture media-derived peptides.

## Discussion

In summary, we present a method for inducing MHC-II in human intestinal enteroids in a physiologically relevant manner. We have shown that IEC MHC-II is controlled by IFNg, a known regulator of MHC-II in IECs *in vivo*, and that IECs possess a functional MIIC, suggesting a role in antigen presentation. Notably, the MHC-II expressed by enteroids was endogenous, in contrast to previous studies in colonic cell lines transfected to overexpress specific MHC-II molecules (32). Because enteroids can be grown from individual patients, they are well-suited for studying the contribution of IEC MHC-II expression to MHC-II-associated diseases, such as celiac disease, in which 95% of patients carry HLA-DQ2 (33). Given the considerable heterogeneity in human populations, patient-to-patient variability in enteroid MHC-II expression is to be expected and was seen in our investigation (Supplementary Figures 2a–c). Further studies are needed to probe genetic and epigenetic sources of patient-to-patient MHC-II variability and may elucidate novel mechanisms of MHC-II regulation.

The ability to maintain MHC-II expression in apical-out or basolateral-out enteroids opens up a wide range of applications. During prolonged intestinal inflammation, the gut epithelium loses integrity, causing IECs to encounter antigen along their basolateral surface. Some studies suggest that IECs process antigens into distinct MHC-peptide epitopes depending on the route of uptake (32). This hypothesis can be readily tested with our protocol and could elucidate the role of breakdowns in epithelial barrier function in intestinal disease. Our polarity reversal approach could also be used to study the effect of enteric pathogens on the MHC-II pathway by infection along the apical surface. For instance, *Salmonella* infection reduces surface levels of MHC-II in human dendritic cells, but such a role has not been explored in the intestinal epithelium, where the pathogen enters (34). Our method therefore lays the groundwork for further investigation of the interactions between IEC MHC-II and the surrounding immune and microbial milieu.

## Data Availability

The datasets generated for this study are available on request to the corresponding author.

## Author Contributions

JW and EM conceived the project and wrote the manuscript, with valuable feedback from DM and all the authors. JW collected and analyzed data, and AI-M assisted with flow cytometry and qPCR data collection and analysis. CK generated enteroids. NF-B provided biopsy tissue for enteroid generation and tissue sections. JC and MA developed the enteroid polarity reversal protocol and provided FITC-Dextran for assessing enteroid barrier integrity. WJ prepared the fluorophore-conjugated Map.DM1 antibody used to detect HLA-DM.

### Conflict of Interest Statement

The authors declare that the research was conducted in the absence of any commercial or financial relationships that could be construed as a potential conflict of interest.
