# Supplementary figures and images for: Human Intestinal Enteroids Model MHC-II in the Gut Epithelium
Source: Front Immunol. 2019 Aug 20;10:1970. doi: 10.3389/fimmu.2019.01970 (PMC6710476; doi:10.3389/fimmu.2019.01970)

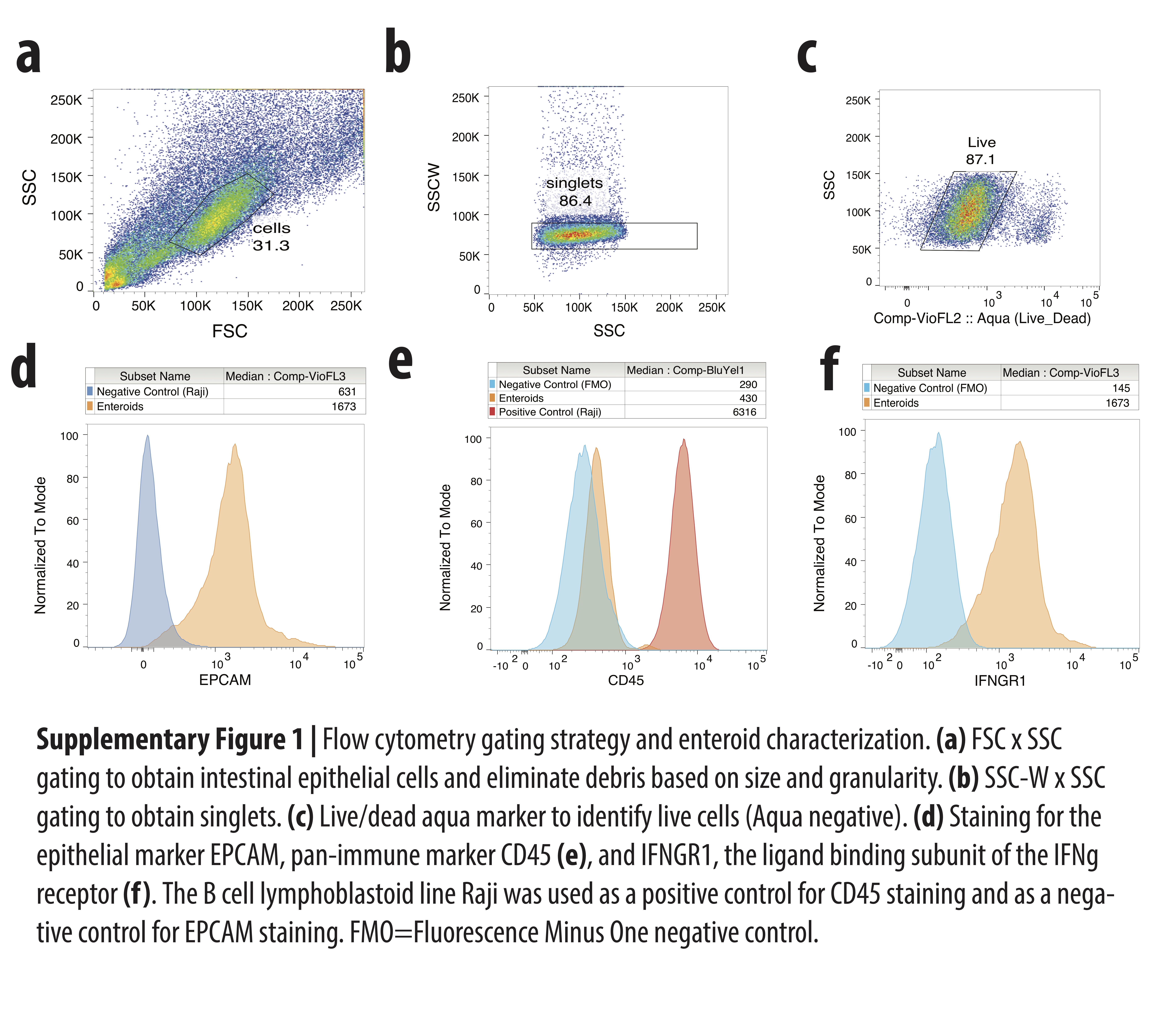

Supplement: Supplementary file 1 [file Image_1.JPEG]

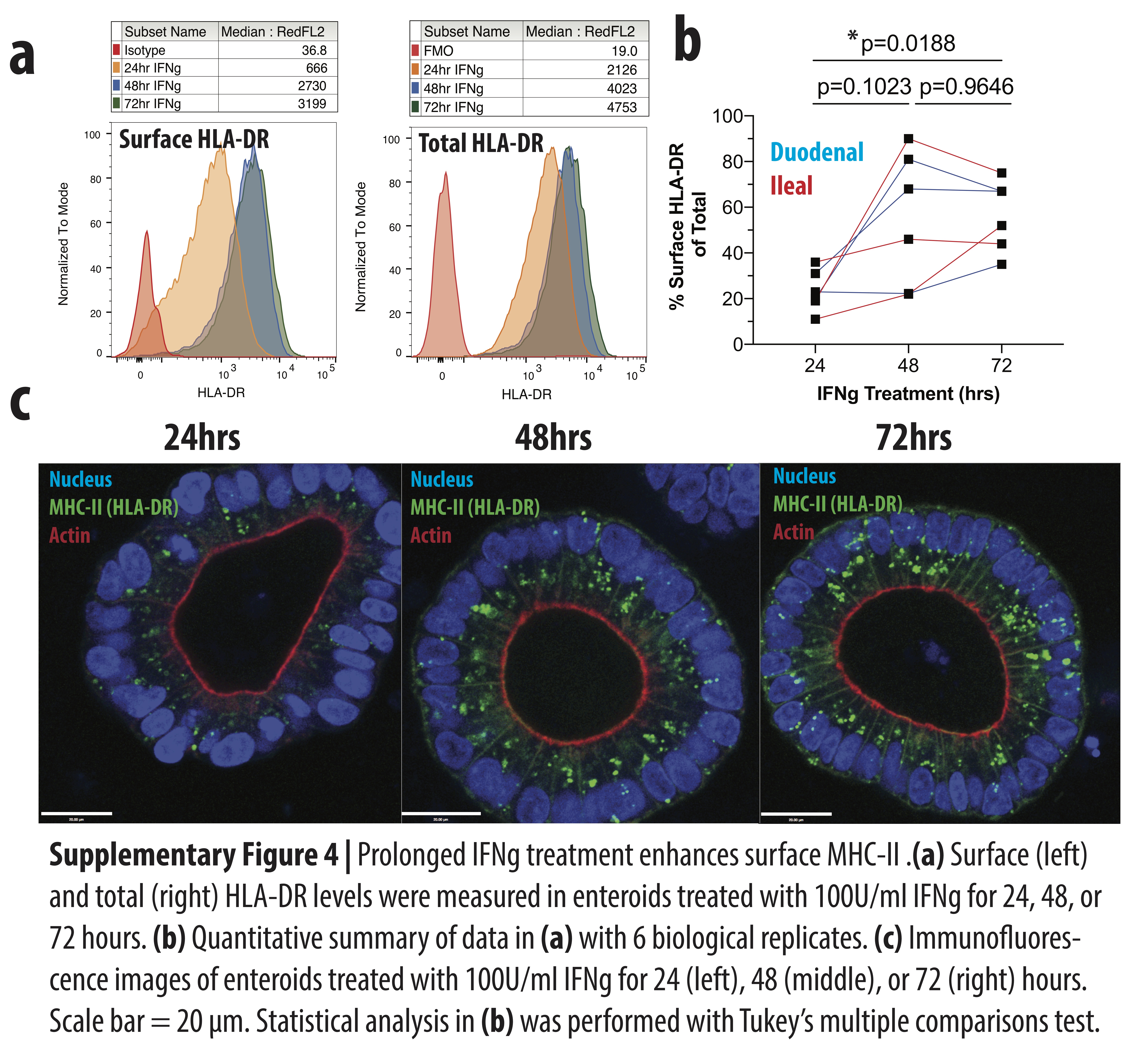

Supplement: Supplementary file 4 [file Image_4.JPEG]

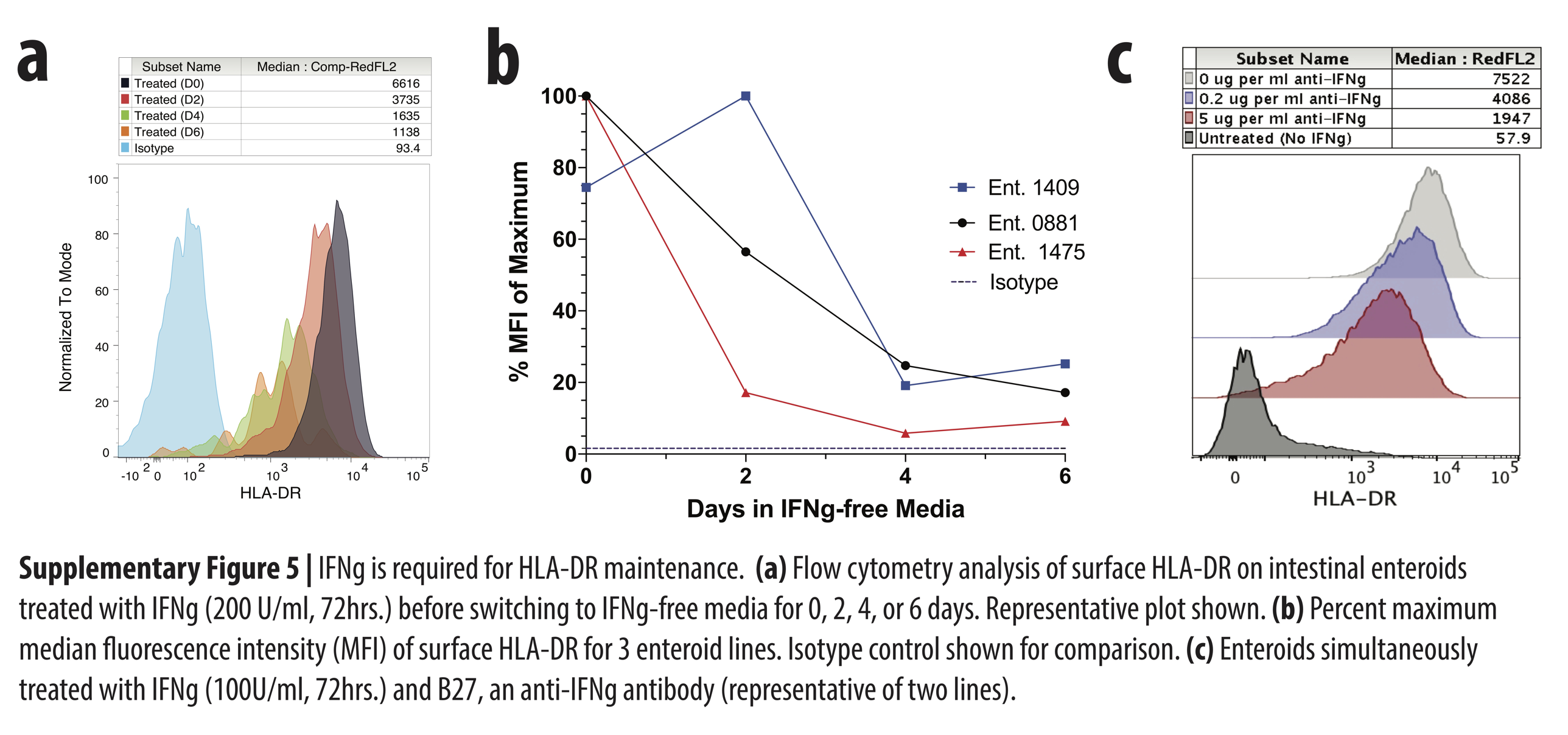

Supplement: Supplementary file 5 [file Image_5.JPEG]

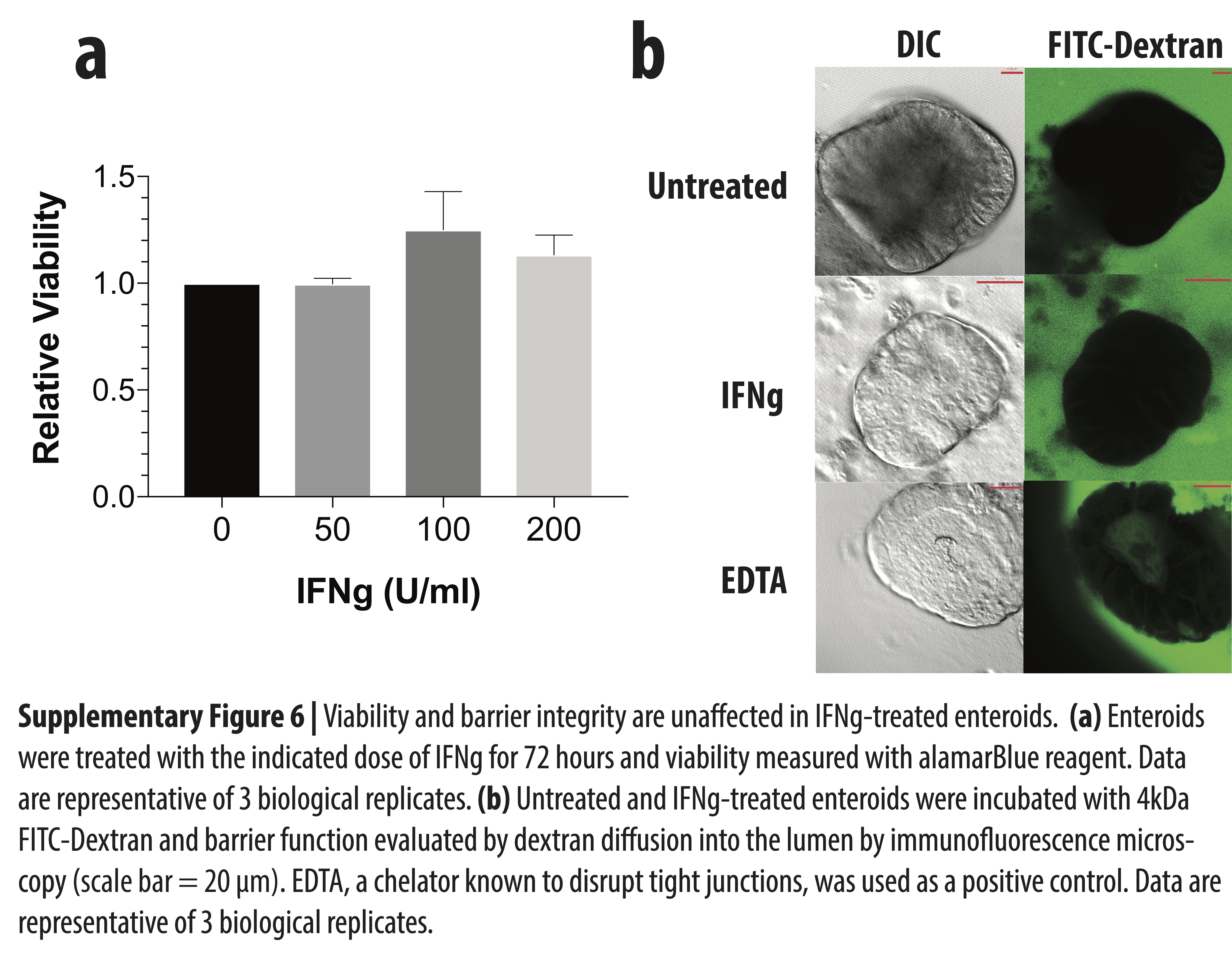

Supplement: Supplementary file 6 [file Image_6.JPEG]

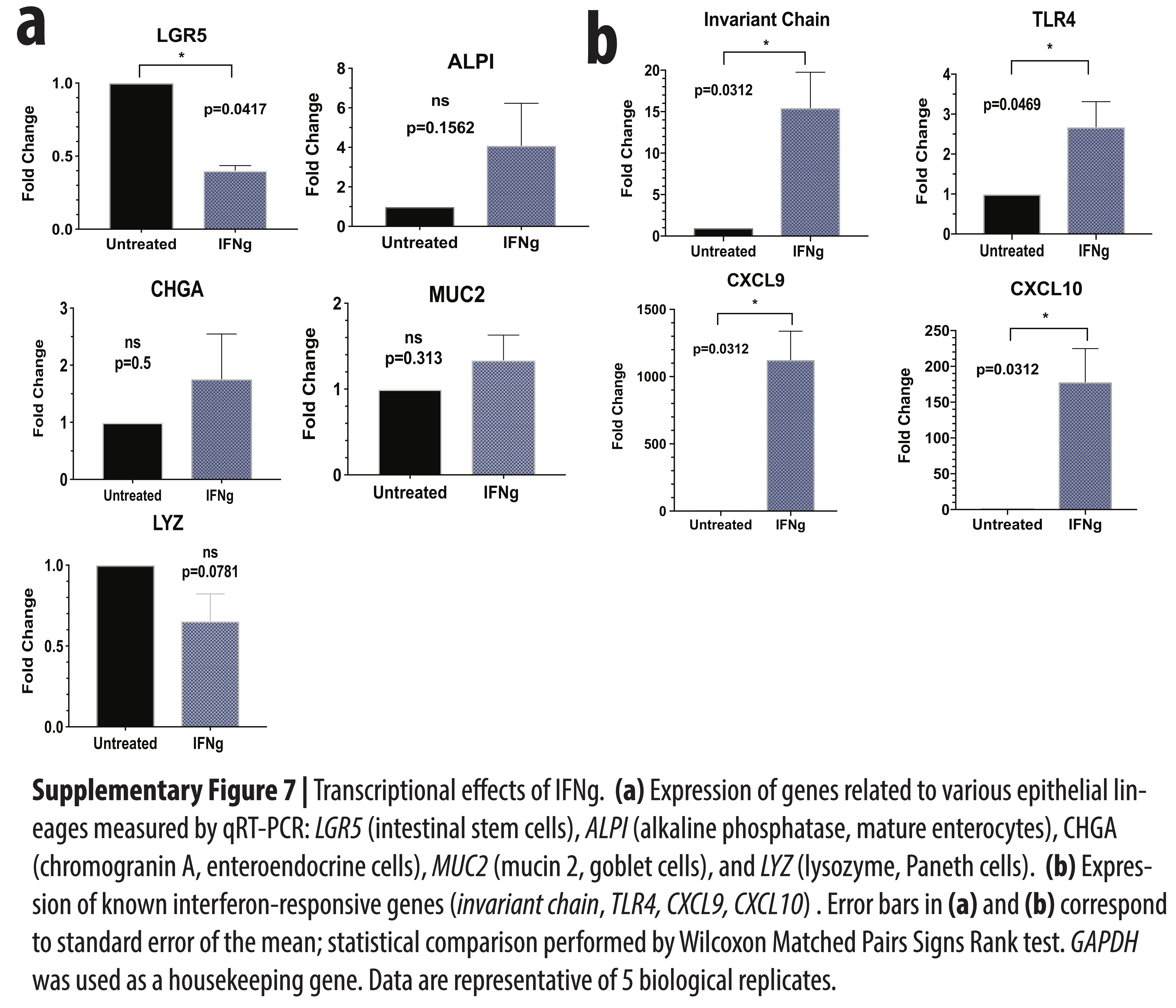

Supplement: Supplementary file 7 [file Image_7.JPEG]

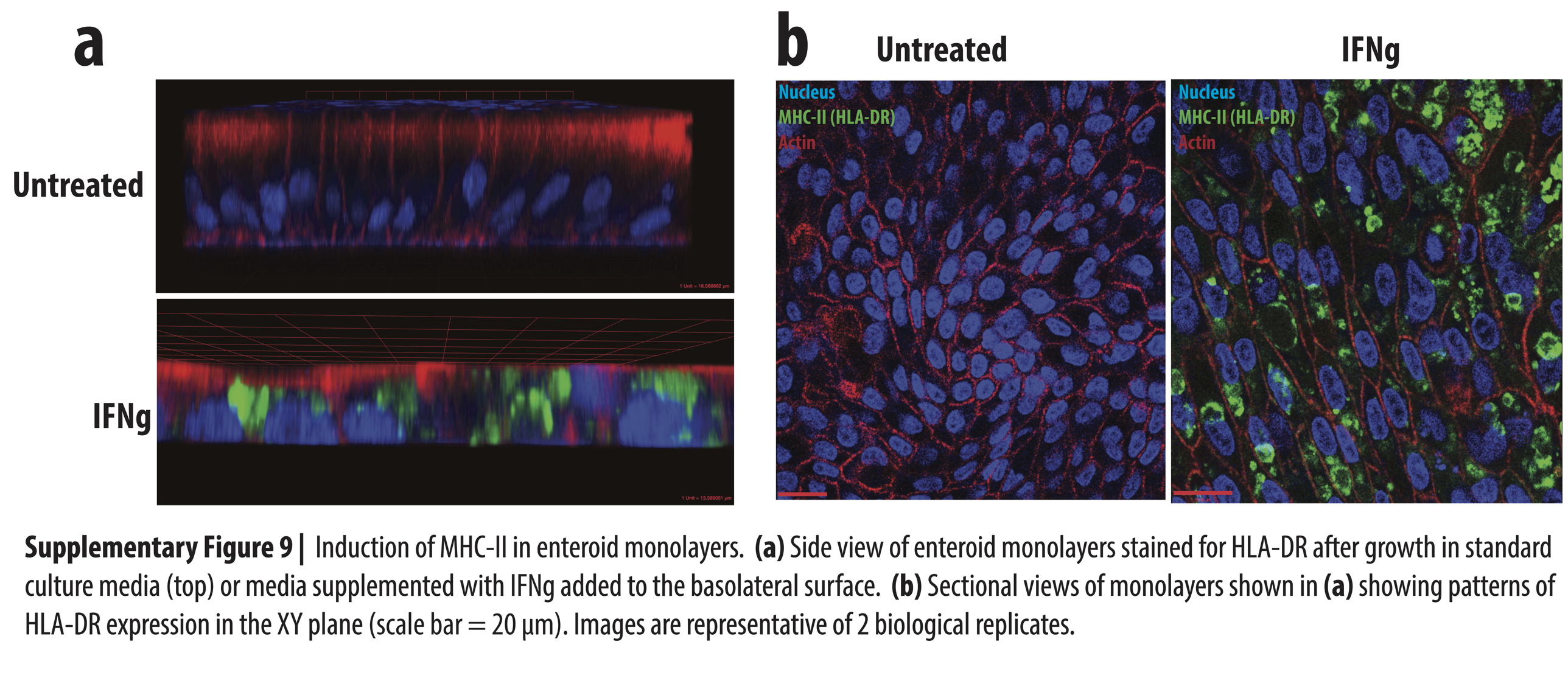

Supplement: Supplementary file 9 [file Image_9.JPEG]

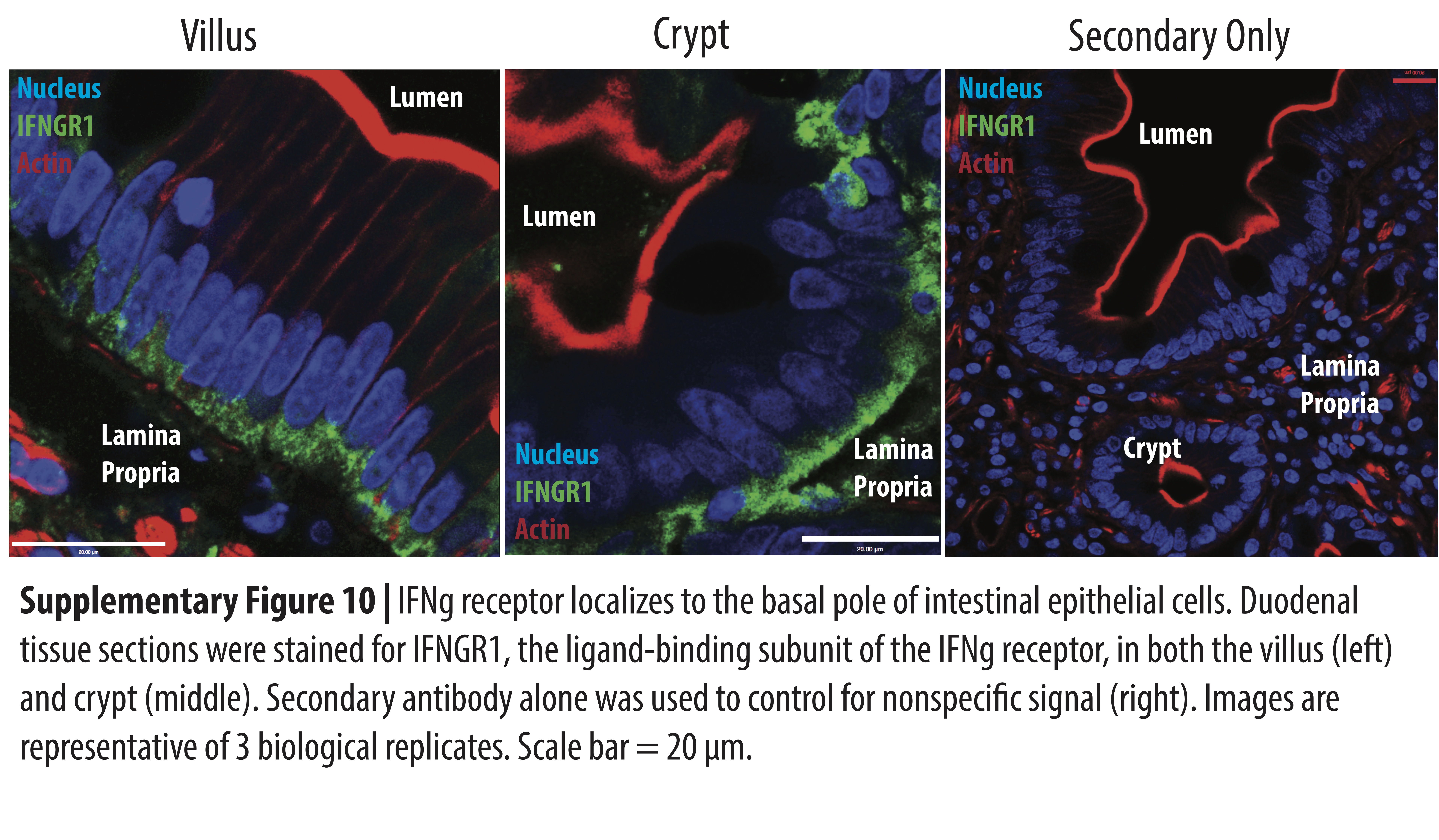

Supplement: Supplementary file 10 [file Image_10.JPEG]
